# The Relation between Gray Matter Morphology and Divergent Thinking in Adolescents and Young Adults

**DOI:** 10.1371/journal.pone.0114619

**Published:** 2014-12-16

**Authors:** Janna Cousijn, P. Cédric M. P Koolschijn, Kiki Zanolie, Sietske W. Kleibeuker, Eveline A. Crone

**Affiliations:** 1 Brain and Development Lab, Department of Psychology, Leiden University, Leiden, The Netherlands; 2 Department of Developmental and Experimental Psychology, Utrecht University, Utrecht, The Netherlands; 3 Dutch Autism & ADHD Research Center, Brain and Cognition, Department of Psychology, University of Amsterdam, Amsterdam, The Netherlands; 4 Leiden Institute for Brain and Cognition, Leiden, The Netherlands; University Children's Hospital Tuebingen, Germany

## Abstract

Adolescence and early adulthood are developmental time periods during which creative cognition is highly important for adapting to environmental changes. Divergent thinking, which refers to generating novel and useful solutions to open-ended problems, has often been used as a measure of creative cognition. The first goal of this structural neuroimaging study was to elucidate the relationship between gray matter morphology and performance in the verbal (AUT; alternative uses task) and visuo-spatial (CAT; creative ability test) domain of divergent thinking in adolescents and young adults. The second goal was to test if gray matter morphology is related to brain activity during AUT performance. Neural and behavioral data were combined from a cross-sectional study including 25 adolescents aged 15–17 and 20 young adults aged 25–30. Brain-behavior relationships were assessed without *a priori* location assumptions and within areas that were activated during an AUT-scanner task. Gray matter volume and cortical thickness were not significantly associated with verbal divergent thinking. However, visuo-spatial divergent thinking (CAT originality and fluency) was positively associated with cortical thickness of the right middle temporal gyrus and left brain areas including the superior frontal gyrus and various occipital, parietal, and temporal areas, independently of age. AUT brain activity was not associated with cortical thickness. The results support an important role of a widespread brain network involved in flexible visuo-spatial divergent thinking, providing evidence for a relation between cortical thickness and visuo-spatial divergent thinking in adolescents and young adults. However, studies including visuo-spatial divergent thinking tasks in the scanner are warranted.

## Introduction

Creativity is generally defined as the generation of new ideas, insights or problem solutions that are original, useful and of a particular level of difficulty [1]. Divergent thinking, which involves the ability to generate new and original solutions to an open-ended problem, is considered a key aspect of creative cognition and recent structural and functional neuroimaging studies have examined the neural areas which support divergent thinking [2]. Divergent thinking involves a complex cognitive process that has been operationalized in different ways. This is reflected in the differential neural findings between studies (for reviews see [2], [3], [4]). One of the currently important questions is whether there are specific neural correlates of divergent thinking, common to different divergent thinking tasks [2]. The current study therefore investigated the relationship between gray matter morphology and divergent thinking in the verbal and visuo-spatial domain in adolescence and early adulthood, as this is a time period during which creative cognition is highly important for adapting to several environmental changes [5].

Divergent thinking tests have commonly been used as a measure of real-life creativity (for a meta-analysis see [6]). A well-known test that measures divergent thinking in the verbal domain is the Alternative Uses Task (AUT; [7]). During the AUT, participants are asked to generate as many ideas as possible about how to use a certain daily life object in an unusual way [8]. AUT performance is generally assessed in terms of fluency (number of ideas), flexibility (number of different conceptual categories in which a solution falls), and originality (uniqueness; [7]). In addition, feasibility (the extent to which a solution can be used in real life) is an important but less measured aspect of divergent thinking [9], [10]. Divergent thinking has also been examined in the visuo-spatial domain using the Creative Ability Test (CAT; [11]). During the CAT, participants view nine squares including a specific pattern of open/closed dots. They are instructed to find as many combinations as possible of three out of the nine squares based on the characteristics of the dots (e.g., color, location, number) without providing information about where solutions can be found and which solutions are required. As such, the AUT and CAT share commonalities because they both require the ability to think of as many new and original solutions in an open-ended task.

Mid-to late adolescence (15–20 years) and early adulthood (20–30 years) may be a specifically important time for divergent thinking. It is well known that during that time adolescents learn to be independent from their parents and adapt to a variety of social situations such as school, university and new working environments [12], [13]. A prior behavioral study showed that performance on both the AUT and CAT improved between late childhood (12–13-years) and mid adolescence (15–16 years) and remained relatively stable between ages 18–19 and 25–30 years. Specifically, 18–19-year-old and 25–30-year old adults had the highest scores on AUT originality, whereas 15–16-year-old adolescents outperformed 12–13-year-old children on the CAT. A model with peak CAT performance in mid adolescence best fitted the data, suggesting an advantage for mid adolescents regarding the visuo-spatial domain of divergent thinking [14]. This may be explained by a more flexible cognitive control system during late adolescence [5], [14]. Yet, prior studies also showed that performances on the AUT and CAT tasks are not highly correlated and load on different factors [15], leading to the question whether the tasks tap into the same or different underlying processes.

On a structural level, adolescent cortical brain maturation tends to follow an ‘‘inverted U’’ developmental course with gray matter volumes peaking at different times in different brain regions [16]. Concurrent cortical thinning occurs throughout adolescence and patterns also differ across brain regions [17], [18]. An important question concerns whether individual differences in divergent thinking are related to individual differences in brain structure, specifically gray matter morphology, as prior studies have reported important relations between brain structure and other domains of cognition [19]–[21]. Few studies investigated the relationship between gray matter morphology and divergent thinking, and the studies to date do not reveal consistent results. For example, performance on the verbal versions of the Torrance test of creative thinking has been associated with larger gray matter volume in bilateral inferior frontal gyri (verbal version of the Torrance test; [22]) whereas performance on the figural version of the Torrance test was associated with a larger right post central gyrus (figural version of the Torrance test; [23]). Second, the total score (composite of fluency, originality and flexibility) on the S-A creativity test of verbal divergent thinking has been associated with larger gray matter volume in right dorsolateral prefrontal cortex, bilateral striatum, dorsal and ventral midbrain and the precuneus [24]. Third, originality of the generated ideas, but not fluency, on the AUT was found to be associated with gray matter density in the right cuneus and occipital regions [25]. Finally, both positive (right posterior cingulate gyrus and right angular gyrus) and negative (left frontal lobe, lingual and angular gyrus, cuneus and inferior parietal cortex) associations were reported with cortical thickness and creativity measures [26]. Taken together, the prior studies do not yet show consistent findings with respect to the brain-behavior associations in the divergent thinking domain of creativity. These discrepant findings may partly be due to the studies' different psychometric measures to assess verbal divergent thinking, as well as different neuroimaging analyses (Voxel Based Morphometry vs. cortical thickness) and different sample characteristics.

One way to derive more hypothesis driven hypotheses to testing brain-behavior relationships is by making use of results of functional neuroimaging studies. That is, functional neuroimaging studies that studied verbal divergent thinking revealed a set of brain regions that are more consistently involved in divergent thinking in adolescence and early adulthood. Using adapted versions of the AUT, researchers have examined which brain areas are more active when thinking of alternative uses relative to thinking about ordinary characteristics of objects [27]–[30]. These studies reported increased activation in the middle temporal gyrus (MTG), angular gyrus, and supramarginal gyrus (SMG) across studies, suggesting that these provide a core neural network for verbal divergent thinking. Functional neuroimaging studies investigating visuo-spatial divergent thinking revealed involvement of partly overlapping, and partly different neural regions compared to what is typically found in verbal divergent thinking studies. One prior study used the figural version of the Torrance Test and showed that performing this task was associated with activity in the posterior parietal cortex, overlapping with the angular gurus and SMG, regions previously associated with verbal divergent thinking [34]. In addition, activity in this region correlated negatively with performance on a visuo-spatial ‘Design a new pen’ task [32]. These results suggest that the neural mechanisms underlying verbal and visuo-spatial divergent thinking may partly overlap in temporal and parietal brain areas. In addition, a prior study showed that visual-spatial divergent thinking, which required the generation of objects from basic shapes, was associated with increased activity in lateral and medial prefrontal cortex, while the posterior parietal cortex and post-central cortex were less active during visuo-spatial divergent thinking relative to a control task [35]. Thus, it remains to be investigated whether verbal and visuo-spatial divergent thinking are associated with the same or different underlying neural regions, specifically in relation to the role of temporal/parietal and frontal brain areas [31], [32].

The first goal of this study therefore was to elucidate the commonalities and differences in the relationship between gray matter volume, cortical thickness and different divergent thinking using tasks tapping into verbal (AUT) and visuo-spatial (CAT) divergent thinking across the brain. For this purpose, behavioral performance during the AUT and the CAT as described by Kleibeuker and colleagues [29] were reanalyzed and related to individual differences in brain structure (i.e., cortical thickness and gray matter volume). In addition to the whole brain analyses, given that the same participants performed a verbal divergent thinking task in the scanner (previously reported in [29]), we reasoned that the brain regions that were activated during verbal divergent thinking were important hypothesis-driven targets for examining the relation between gray matter morphology and both verbal and visuo-spatial divergent thinking task performance. Even though this approach was limited to the verbal domain of divergent thinking, the region of interest (ROI) selection based on this verbal task provides a more in-depth complementary analysis for examining differences and commonalities between brain regions important for verbal and visuo-spatial divergent thinking.

Finally, Kleibeuker and colleagues [29] measured both behavioral performance and brain functioning during the AUT task in middle adolescents aged 15–17 and young adults aged 25–30. Therefore, this dataset provided the unique opportunity to assess the associations between gray matter morphology and functional brain activity associated with verbal divergent thinking. The second goal therefore was to investigate if cortical thickness is associated with BOLD activity in the regions activated during performance of the AUT in the scanner. Given the strong relationship between age and gray matter morphology, it was also investigated if age group (adolescents vs. adults) moderated the relationship between divergent thinking (on a behavioral and neural level) and gray matter morphology. We predicted that cortical thickness in the main brain areas activated during the AUT would be positively related to AUT behavioral scores and AUT brain activity, irrespectively of age under the assumption that neural structure is an underlying factor that aids in task performance [31].

## Materials & Methods

This study was part of a large project investigating the neural mechanisms underlying creative cognition [14], [29], [32]. All procedures were approved by the Internal Review Board of Leiden University Medical Center (LUMC).

### Participants

Table 1 provides an overview of the sample characteristics. A total of 25 adolescents and 20 adults were included in the analyses. Sex distribution (χ^2^ = .04, *p* = .84) and estimated IQ scores (*t*_43_ = .73, *p* = .47) derived from the Wechsler Adult Intelligence Scale (WAIS; [33]) Digit Span and Similarities subtests did not differ between age groups. All participants were right-handed, had normal or corrected to normal vision, no contraindications for MRI, and no self-reported history of neurological or psychiatric disorders. Participants were recruited from the general population trough local advertisements. All participants provided written informed consent. In case of minors, written consent was also obtained from their primary caregivers. Participation was compensated with either money or course credits.

**Table 1:** Sample characteristics.

	Adolescents	Adults
N (males)	25 (12)	20 (9)
Age	16.9 (0.6)	26.9 (1.4)
IQ	111.1 (6.7)	113.6 (15.1)
AUT-scanner fluency	2.3 (0.6)	2.7 (0.6)
AUT-brick fluency^1^	8.6 (4.6)	12.7 (5.6)*
AUT-brick flexibility^1^	6.7 (2.8)	7.9 (2.7)
AUT-brick originality^1^	1.7 (0.4)	1.6 (0.4)
AUT-brick feasibility^1^	4.4 (1.0)	4.7 (0.3)
CAT fluency^2^	9.8 (2.3)	9.0 (2.6)
CAT originality^2^	15.5 (5.8)	14.7 (7.1)
TIV	1635.4 (167.1)	1522.9 (192.8)*

Means (standard deviation) are depicted. *p<0.05 for group comparisons. AUT; Alternative Uses Test. CAT; Creative Ability Test. TIV; Total Intracranial Volume. ^1^AUT-brick data missing of one adolescent and two adults, ^2^CAT data missing from one adolescent.

### Measures of divergent thinking

#### Alternative Uses Test-scanner (AUT-scanner)

All participants performed an adapted version of the Alternative Uses Test (AUT; [34]) inside the MRI scanner (see [29], for a detailed task description]. This task measures divergent thinking in the verbal domain. In short, the task consisted of an Alternative Uses (AU) condition and an Object Characteristics (OC) condition. During AU trials participants had to think of as many appropriate alternative and original uses of common objects as possible (i.e., use a shoe as a baseball bat). During OC trials participants had to think of as many ordinary characteristics of common objects as possible (i.e., a shoe fits on a foot). Each trial started with a 3 seconds instruction screen, followed by the target screen that lasted for 15 seconds. Immediately after the target screen, the participants had 3 seconds to indicate the number of generated solutions by pressing a response box button corresponding to ‘0–1’, ‘2’, ‘3’ or ‘4+’. Each trial was preceded by a fixation cross with a jittered duration (0–6 seconds). The AUT-scanner consisted of 40 trials divided over two blocks, using 40 unique words (20 words presented in the AU and 20 words in the OC condition). AUT-scanner performance was measured by calculating the average number of solutions (fluency).

#### Alternative Uses Test-brick (AUT-brick)

All participants performed the AUT-brick task outside of the scanner (Friedman & Forster, 2001). The AUT-brick is a single item version of the AUT, similar to the AUT-scanner. The AUT-brick measures divergent thinking in the verbal domain and performance on the AUT-scanner and AUT-brick is strongly related [29]. Participants were instructed to generate as many appropriate alternative and original uses for a brick. Participants typed their solutions one at the time on a laptop during a period of four minutes (for similar procedure see for example [35], [36]). An experienced independent rater (TdW) rated the given solutions based on the scoring algorithm developed and validated by [37], [38]. The rater was first extensively trained until high inter-rater reliabilities for originality scores were achieved (ICC = 0.91, based on AUT-Brick data which was concurrently rated for a different independent study with similar age-groups [39]). *Fluency* scores were computed by counting the number of correct solutions provided. *Flexibility* scores were computed by counting the number of different solution-categories. The independent trained rater assigned each solution to one of 35 predefined solution-categories (i.e., building aspect, load, toy; [37], [38]). The number of different applied solution-categories was subsequently summed per participant. *Originality* was assessed on a 5-point scale (from 1 =  “not original” to 5 =  “highly original”). Originality scores were computed by averaging the ratings of all solutions per participant. *Feasibility* scores were computed by scoring the feasibility and usefulness of the generated solutions. The feasibility scores of the generated solutions were subsequently summed per participant. Participants were not informed about how their performance would be rated.

#### Creative Ability Test (CAT)

All participants performed the Creative Ability Test (CAT; [40]), a creativity task which measures divergent thinking in the visuo-spatial domain (test-retest reliability: r = .69). It is a pencil and paper task during which the participants view nine squares including dots. Per square, the dots can differ in location, color and number. Participants were instructed to find triads of squares based on the characteristics of the dots (i.e., number, color, location). *Fluency* scores were computed by counting the number of correct solutions provided. *Originality* scores were computed by summing the uniqueness score of each correct answer. Uniqueness (1 =  ‘common’ to 5 =  ‘very unique’) was determined by the occurrence of a given solution in previous validation studies [11]. The response time was limited to 10 minutes.

### MRI Data acquisition

Scanning was performed with a standard 32-channel whole-head coil on a 3-T Philips Achieva MRI system in the Leiden University Medical Center. High-resolution 3D T1-weighted anatomical scans were acquired (TR = 9.717 ms; TE = 4.59 ms, flip angle  = 8 degrees, 140 slices, 0.875×0.875×1.2 mm, field of view  = 224.000×168.000×177.333). In accordance with Leiden University Medical Center policy, all anatomical scans were reviewed and cleared by a radiologist from the Radiology department. Bold signal during the AUT-scanner was measured with a T2* gradient-echo EPI sequence (TR 2.2s, TE 30 ms, 38 slices, slice thickness 2.75 mm, FOV 220×220 mm, in-plane resolution 2.75×2.75 mm, flip angle 80°, sequential slice acquisition [29].

### MRI Image analyses

#### Cortical reconstruction

Cortical reconstruction was measured automatically using FreeSurfer version 5.3 (http://surfer.nmr.mgh.harvard.edu/, [41], [42]. Details of the surface-based cortical reconstruction and subcortical volumetric segmentation procedures have been extensively documented previously [41]–[44]. Briefly, the FreeSurfer pipeline performs motion correction on the T1-images, automatically removes non-brain tissues [44], transforms volumetric data to a common atlas, performs intensity normalization and topology correction [43], [45] and defines the boundaries of the gray/white matter and pial surface [41], [42]. For the purposes of the current study, automated image surfaces and segmentations were inspected and screened for quality control but were not manually edited, in order to maintain the objectivity of results. No scans were excluded due to poor quality or poor segmentations. Total intracranial volume (TIV) was determined by a validated automated method known to be equivalent to manual intracranial volume estimation [46].

#### Region of Interest definition (ROI)

**Figure 1:**
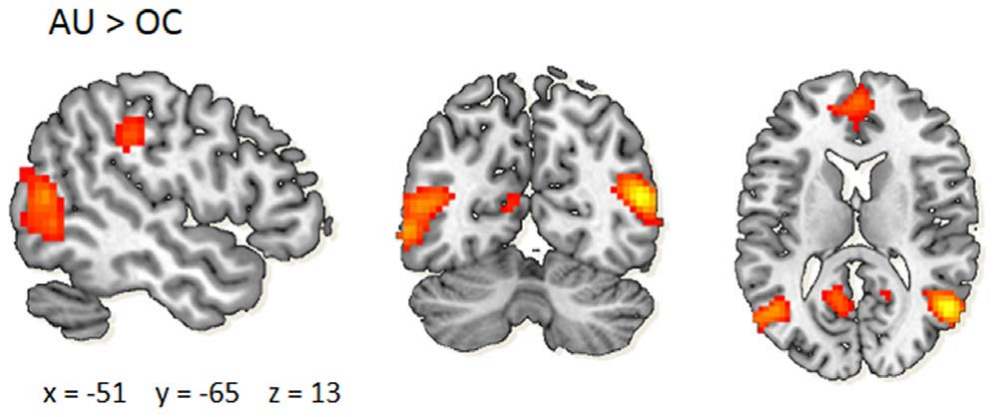
Neural activations during the alternative uses task (AUT). Main effect of AU > OC: left Supramarginal Gyrus, left Inferior Parietal Cortex, bilateral Anterior Cingulate Cortex, right Postcentral Gyrus, right Middle Temporal Gyrus. Statistical threshold >100 contiguous voxels, *p*<0.05 FDR-corrected for multiple comparisons. AU; alternative uses. OC; ordinary characteristics.

The neural results of this task in the present sample were recently published [29]. Brain activity during AU>OC across age groups (FDR-corrected p<.05 and at least 10 contiguous voxels) was used to determine the ROIs included in the present analyses (Fig. 1). The MarsBar toolbox for SPM [47] was used to extract the ROIs from the main contrast and average BOLD activity was extracted from each ROI. ROIs were included in the current analyses if the activated area consisted of >100 contiguous voxels, to ensure a meaningful anatomical interpretation. A total of five ROIs were thereby identified (Table 2): left SMG, left Inferior Parietal Cortex (IPC), bilateral Anterior Cingulate Cortex (ACC), right Postcentral Gyrus (PG), and right MTG. Average beta-values were extracted from each ROI for the contrast AU > OC to use as a neural measure of AUT performance. To extract average cortical thickness from the functional ROIs, we performed the following steps: 1) Each functional ROI was registered automatically to the FreeSurfer “fsaverage” template using SPM's coregistration (spm_coreg) with Normalized Mutual Information and inspected for accuracy of registration. Of note, as FreeSurfer calculates cortical thickness per hemisphere, the ACC ROI was split into a left and right structural ROI. 2) Individual cortical thickness data was mapped to the “fsaverage” template. 3) Average cortical thickness in mm was extracted for each ROI and individual separately. 4) For correlation analyses, the bilateral ACC ROI was averaged back to one ROI. We used an average weighted procedure by taking into account hemispherical differences in surface size maps.

**Table 2:** Region of Interest size and activity during the AUT-scanner as reported in Kleibeuker, et al., 2013b.

Brain area	Side	MNI coordinates			Size (voxels)	*Z*_max_
		x	y	z		
Supramarginal Gyrus	left	−60	−30	36	189	6.63
Inferior Parietal Cortex	left	−42	−84	30	265	5.01
Anterior Cingulate Cortex	left/right	−3	48	0	834	4.91
Postcentral Gyrus	right	36	−30	51	199	4.73
Middle Temporal Gyrus	right	48	−63	15	160	4.49

MNI coordinates and maximum *Z*-value are given for AU > OC across adults and adolescents. All regions survive FDR-correction *p*<.05, >100 contiguous voxels. MNI; Montreal Neurological Institute

### Statistical analyses

#### Behavioral analyses

Behavioral data were analyzed using standard analyses of variance (ANOVAs) and t-tests. Post-hoc t-test were Bonferroni corrected (indicated as *p*_corr_) for multiple comparisons. Analyses were conducted with SPSS (IBM SPSS Statistics for Windows, Version 20. Armonk, NY: IBM Corp.).

#### Whole brain vertex-wise analyses

The cortical thickness data were averaged across the whole study sample in the spherical coordinate system after smoothing (FWHM 15 mm), so that surface areas with a significant effect of mean cortical thickness differences and the different creativity measures could be overlaid in statistical difference maps (using *t*-statistics) for both the cross-sectional and training study. To investigate the relationship between divergent thinking and gray matter morphology, a series of vertex-wise analyses looking both at cortical thickness or gray matter volume were performed using a general linear model approach in FreeSurfer. We investigated 1) the linear relationship between all divergent thinking measures (AUT-scanner, AUT-brick, CAT) and brain structure across age groups, and 2) the moderating effect of age group on the relationship between divergent thinking and gray matter morphology. All analyses were controlled for age, sex and TIV. A continuous discussion in the field of creativity research is the influence of IQ on creative abilities. Although prior studies indicate a modest relationship between IQ and divergent thinking tasks [6], [48], [49], we ran subsequent analyses with IQ as a covariate to rule out IQ-related effects. Differences were reported as significant below a FDR corrected *p*-value of 0.05.

#### Region of Interest Analyses

Within each ROI, a similar series of analyses were performed to investigate the relationship between divergent thinking (behavioral performance during the AUT-scanner, AUT-brick, CAT, and brain activity during the AUT-scanner) and cortical thickness. All analyses were controlled for age, sex and TIV. Although the functional ROIs were based on group level analyses and hence, ROI extent is similar for each individual, it is important to correct for TIV. In the functional analyses, a larger brain may lead to a relatively smaller ROI (on group level), and a smaller brain to a relatively larger ROI (based on group level). We investigated 1) the linear relationship between cortical thickness and behavioral divergent thinking, 2) the moderating effect of age group on the relationship between cortical thickness and behavioral divergent thinking, 3) the linear relationship between cortical thickness and BOLD activity for the AU > OC contrast during the AUT-scanner, and 4) the moderating effect of age group on the relationship between cortical thickness and BOLD activity. To investigate the moderating effects, regression models were built entering sex, TIV, age group (dummy coded) and the measure of divergent thinking (behavior or BOLD activity) in step one, after which the interaction term between age group (dummy coded) and the measure of divergent thinking was entered. In subsequent analyses, the same procedure was applied with IQ as an additional factor in step one of the regression model. The ROI-analyses were Bonferroni-corrected for the number of ROIs, resulting in a critical *p*-value of 0.01. Analyses were conducted with SPSS for Windows (v.20).

## Results

### Behavioral analyses

The behavioral findings from the cross-sectional study were published previously [29]. In short, AUT-scanner fluency scores were not significantly higher for adults compared to adolescents (*t*_43_ = 1.76, *p* = .085). AUT-brick data were missing for one adolescent and two adults. For AUT-brick fluency, adults performed significantly better than adolescents (*t*_39_ = 2.36, *p* = .023), whereas flexibility (*t*_39_ = 1.49, *p* = .15), originality (*t*_39_ = 1.24, *p* = .22), and feasibility (*t*_39_ = 1.17, *p* = .25) did not differ between age groups. CAT data were missing for one adolescent. CAT fluency and originality did not differ between adolescents and adults (*t*_42_ = 1.14, *p* = .26 and *t*_42_ = .39, *p* = .70, respectively).

### Whole brain vertex-wise analyses

**Figure 2:**
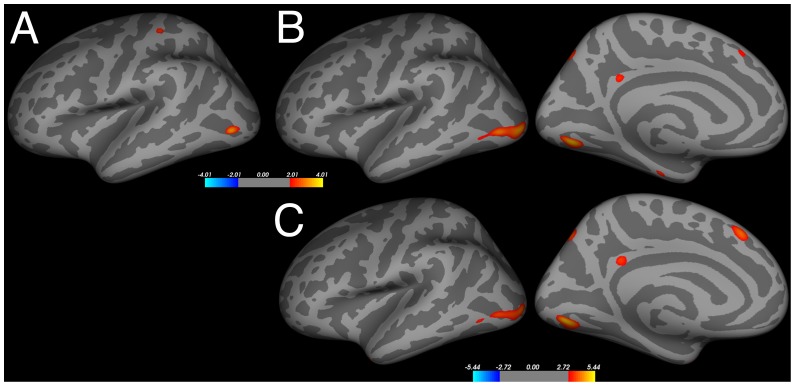
The association between performance on the visuo-spatial divergent thinking task (CAT) and cortical thickness observed in the whole-brain analyses. (**A**) Positive association with *CAT fluency.* (**B**) Positive association with *CAT originality*. Note, TIV, age and sex were used as nuisance variables, FDR-corrected, *p*<0.05 in both figures. (**C**) Positive association with *CAT originality corrected for IQ*. Note, TIV, age and sex were used as additional nuisance variables, FDR-corrected, *p*<0.05 in all figures. Hot colors indicate cortical thickening, cool colors thinning.

There were no significant relationships between any of the verbal divergent thinking measures (AUT-scanner, AUT-brick) and cortical thickness or gray matter volume. Neither age-group nor sex was a significant moderator in any of these analyses (including TIV, age, sex (for age-group comparisons) as nuisance factors). Performance on the visuo-spatial divergent thinking task (CAT) was associated with cortical thickness in discrete brain areas (Table 3 and Fig. 2A). Specifically, positive associations were found between CAT-fluency and cortical thickness of the left lateral occipital cortex and postcentral gyrus. These associations were no longer significant after IQ correction. Moreover, CAT-originality was positively associated with cortical thickness of the lateral occipital cortex, lingual gyrus, superior parietal cortex, temporal pole, enthorinal cortex, isthmus cingulate and superior frontal cortex, irrespective of IQ correction (Fig. 2B, 2C). No significant associations were found between CAT performance and gray matter volume. Moreover, neither age-group nor sex was a significant moderator of the relationship between CAT performance and cortical thickness or gray matter volume.

**Table 3:** Relationship between cortical thickness and Creative Ability Test performance.

Measures of divergent thinking	Brain area	Side	Talairach coordinates*			Size(mm^2^)	*t*_max_
			x	y	z		
*CAT fluency* 1							
	Lateral Occipital Cortex	left	−41.2	−75.2	−2.5	118.2	5.48
	Postcentral Gyrus	left	−37.5	−33.7	62.2	46.4	4.61
*CAT originality*							
	Lingual Gyrus	left	−18.7	−75.8	−9.2	398.0	5.03
	Lateral Occipital Cortex	left	−28.4	−94.0	−5.2	753.7	4.67
	Superior Parietal Cortex	left	−13.0	−76.4	43.7	78.7	3.90
	Temporal Pole	left	−32.7	8.9	37.7	84.8	3.87
	Enthorinal Cortex	left	−27.8	−17.4	−30.6	49.3	3.63
	Isthmus Cingulate Cortex	left	−5.4	−45.1	30.7	36.2	3.60
	Superior Frontal Gyrus	left	−7.8	29.5	45.3	25.0	3.36
*CAT originality with IQ*							
	Lingual Gyrus	left	−15.5	−78,9	−10.8	484.5	5.00
	Temporal Pole	left	−33.0	10.5	−38.0	143.7	4.21
	Lateral Occipital Cortex	left	−28.5	−94.4	−3.0	580.2	4.16
	Superior Frontal Gyrus	left	−7.4	28.4	45.8	130.4	4.15
	Superior Parietal Cortex	left	−12.5	−76.2	43.5	105.0	4.02
	Isthmus Cingulate Cortex	left	−4.3	−43.2	29.7	53.9	3.72
	Inferior Temporal Cortex	left	−47.3	−63.6	−5.4	20.6	3.46

*t*_max_ = *t-*value of maximum positive correlation between cortical thickness and CAT performance. All regions survive FDR-correction *p*<0.05 with Total Intracranial Volume, sex and age as nuisance variables. Size (mm^2^)  =  surface area of the cluster in square millimeters.*Note on Talairach: FreeSurfer does not report true "Talairach" coordinates. The coordinates listed under "Talairach" are actually based on Matthew Brett's 10/8/98 non-linear transform from MNI305 space (see https://surfer.nmr.mgh.harvard.edu/fswiki/CoordinateSystems).

1 *No significant associations after IQ correction.*

### Region of Interest Analyses

**Figure 3:**
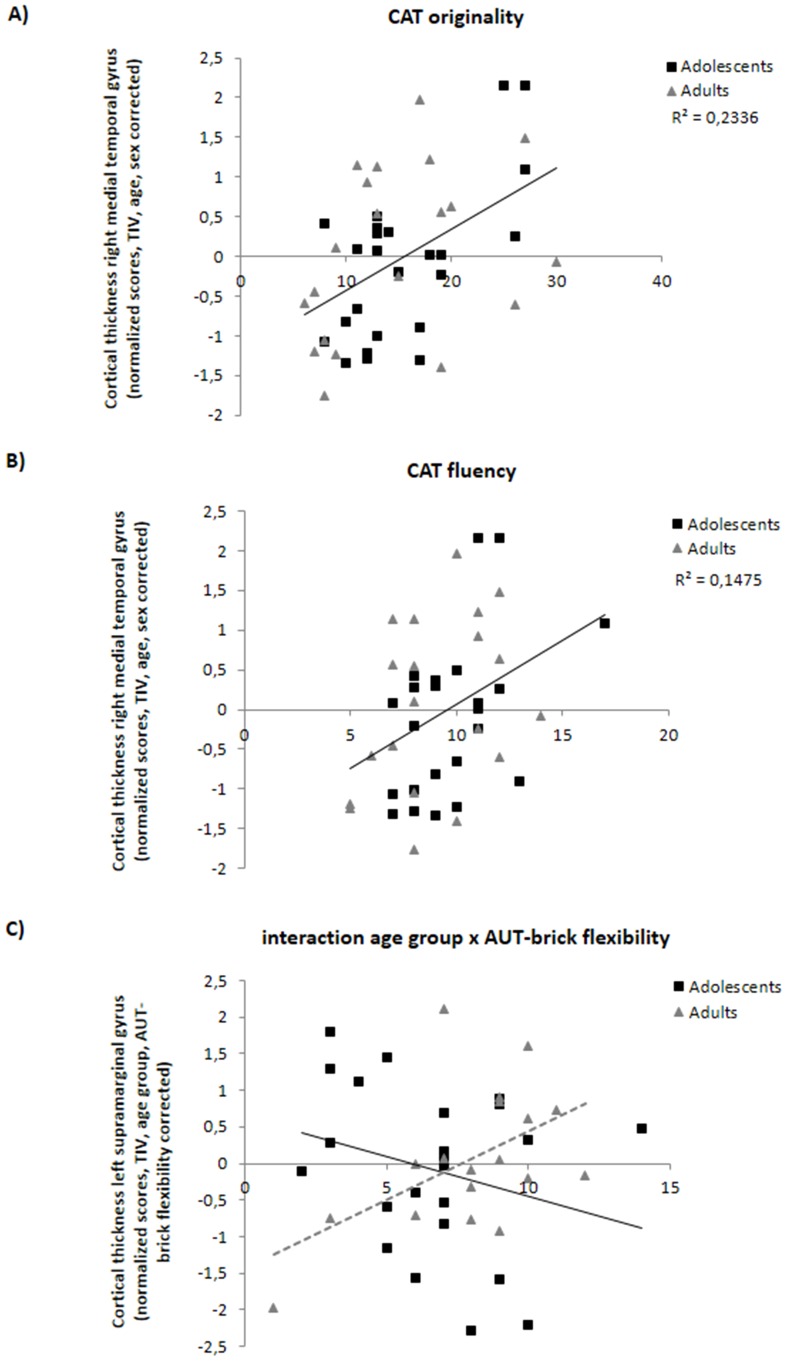
The association between performance on the divergent thinking tasks and cortical thickness observed in the ROIs. Positive association between cortical thickness of the right Middle Temporal Gyrus and (**A**) CAT originality and (**B**) CAT fluency. (**C**) Moderating effect of age group on the relationship between cortical thickness of the left Supramarginal Gyrus and AUT-brick flexibility.

A series of hierarchical regression analyses with TIV, age, and sex entered in the first step and a measure of divergent thinking (AUT-scanner, AUT-brick, CAT) in the second step were conducted to investigate the linear relationship between cortical thickness in the five ROIs (SMG, IPC, ACC, PC MTG) and divergent thinking. CAT originality was positively related to cortical thickness of the right MTG (Fig. 3A). Total variance explained by this model was 48% (*F*_4,39_ = 9.00, *p*<.001) with no violation of the assumption of normality, linearity, multicollinearity, and homoscedasticity (maximum Cook's distance  = .23, maximum standardized residual  = 2.80). The control variables in step 1 TIV, age, and sex explained 31% of the variance in cortical thickness of the MTG, with age being a significant predictor (*p*<.001). CAT originality explained an additional 17% of the variance (*F*_1,39_ = 13.07, *p* = .001, ß = .43): higher originality was related to larger cortical thickness of the right MTG. Similarly CAT fluency was positively related to cortical thickness of the right MTG (Fig. 3B). Total variance explained by this model was 42% (*F*_4,39_ = 7.14, *p*<.001) with no violation of the assumption of normality, linearity, multicollinearity, and homoscedasticity (maximum Cook's distance  = .12, maximum standardized residual  = 1.97). After correction for TIV, age, and sex in step 1, CAT fluency explained an additional 12% of the variance (*F*_1,39_ = 7.89, *p* = .008, ß = .36): higher fluency was related to larger cortical thickness of the right MTG. No other significant relationships were observed between cortical thickness and divergent thinking.

Age group significantly moderated the relationship between AUT-brick flexibility and cortical thickness of the left SMG (Fig. 3C). Total variance explained by this model was 40% (*F*_5,39_ = 5.14, *p* = .001) with no violation of the assumption of normality, linearity, multicollinearity, and homoscedasticity (maximum Cook's distance  = .24, maximum standardized residual  = 2.27). The control variables in step 1 TIV, age group, sex, and AUT-brick flexibility explained 28% of the variance in cortical thickness of the SMG, with age group being a significant predictor (*p*<.001). The interaction term between age group and AUT-brick flexibility explained an additional 12% of the variance (*F*_1,39_ = 7.79, *p* = .008, ß = −.51). The moderation effect indicated a significant negative relationship between cortical thickness of the SMG and AUT-brick flexibility in the adolescents only. Age group did not significantly moderate the relation between cortical thickness of the other ROIs and any of the other measures of divergent thinking. Moreover, there was no significant linear relationship between cortical thickness and BOLD activity for the AU > OC contrast during the AUT-scanner in any of the ROIs when correcting for age, sex and TIV. Finally, age group did not significantly moderate the relation between cortical thickness BOLD activity for the AU > OC contrast.

All ROI analyses were run a second time with an additional correction for IQ. Results and interpretations remained similar: CAT fluency explained 14% of the variance (F_1,38_ = 10.08, p = .003, ß = .40) in cortical thickness of the right MTG. CAT originality explained 22% of the variance (F_1,38_ = 18.33, p<.001, ß = .50) in cortical thickness of the right MTG. The interaction between age group and AUT-brick flexibility explained an additional 15% of the variance (F_1,38_ =  9.81, p = .003, ß = −.59) in cortical thickness of the left SMG.

## Discussion

The first goal of this structural neuroimaging study was to elucidate commonalities and differences in the relationship between gray matter morphology and performance in the verbal (AUT) and visuo-spatial (CAT) domain of divergent thinking in middle adolescents and young adults. These relationships were assessed across the brain without *a priori* location assumptions and within ROIs that were activated during a verbal divergent thinking task [29], e.g., the left SMG, left IPC, bilateral ACC, right PC and right MTG. The second goal was to test if cortical thickness in the ROIs was associated with functional brain activity during verbal divergent thinking.

Gray matter morphology in terms of volume and cortical thickness was not significantly associated with performance on the AUT. However, whole-brain analysis showed that CAT performance (i.e., originality and fluency) was positively associated with cortical thickness of left brain areas including the superior frontal gyrus and various occipital, parietal, and temporal areas. Moreover, ROI analysis indicated that higher CAT originality and fluency were related to larger cortical thickness of the right MTG. These results suggest a positive relationship between cortical thickness and divergent thinking in the visuo-spatial, but not the verbal domain. Moreover, age group moderated the relationship between AUT flexibility and cortical thickness of the left SMG, showing a negative relation between cortical thickness and flexibility in adolescents only. The discussion is organized along the lines of these main findings.

### Gray matter morphology in relation to divergent thinking

Gray matter morphology is thought to be an underlying factor that aids task performance [31], [50]–[52]. Regarding AUT performance, a previous VBM study conducted in a large sample (N = 71) of young adults (university students) found that gray matter density of the right occipital cortex and cuneus was positively related to AUT originality scores (but not fluency) [25]. The authors suggested that this structural association represents a functional association between visual mental imagery and divergent thinking. In contrast to Fink and colleagues [25], we did not observe such a relationship between originality scores on the single item AUT-brick and cortical thickness or gray matter volume in late adolescents and young adults. These inconsistent findings may, at least in part, be attributed to differences in methodology (differences in sample size, VBM vs. cortical thickness and single vs. multi-item AUT). Nonetheless, these findings also suggest that the relationship between gray matter morphology and verbal divergent thinking as measures with the AUT may be relatively weak or subject to individual differences, warranting large sample sizes in the study of the neurobiology underlying creative cognition.

We showed that a visuo-spatial measure of divergent thinking was positively related to cortical thickness of various brain areas. Cortical thickness of the left occipital cortex and right MTG were associated with both CAT fluency and originality. CAT originality and fluency share some variance as the more original solutions often appear after more common answers, however, originality is thought to reflect real life creativity more strongly than fluency [37]. Cortical thickness of the left postcentral gyrus was specifically related to CAT fluency, whereas CAT originality was specifically associated with various left temporal, parietal, and frontal areas. These structure-function associations converge largely with brain activity of non-verbal creativity tasks from a recent meta-analysis of fMRI studies [53]. The MTG, an important area underlying verbal divergent thinking [29], [54], showed a significant association with CAT originality and fluency in the ROI analysis. In the whole-brain analysis, this cluster did not survive whole-brain multiple comparison correction; therefore the effect should be replicated in future studies. It should be noted that we couldn't determine whether performance on the CAT would have resulted in functional brain activity in the same brain areas in which we found an association between CAT performance and cortical thickness. CAT performance data was collected outside the MRI-scanner only, which is a limitation. To fully understand the common and unique neural mechanisms underlying verbal and visuo-spatial divergent thinking, future studies should include both types of tasks in a scan-session. Nonetheless, this result may suggest that right MTG involvement is not limited to verbal divergent thinking as demonstrated by previous fMRI studies [29], [54] and resting-state studies [55], but may be more generally involved in divergent thinking.

Interestingly, the (predominately left) areas in which a positive relationship was observed between CAT performance and cortical thickness are known to play a role in the processing of complex visual information (e.g., lateral occipital cortex, lingual gyrus; [56], [57]), spatial memory (enthorinal cortex; [58]), semantic memory (temporal pole; [59]), episodic memory retrieval and attention (isthmus of cingulate cortex; [60]), and working memory (superior frontal gyrus, superior parietal gyrus; [61]). In general, the right hemisphere may be specialized for visuo-spatial processing compared to the left hemisphere [62]. However, in creativity-related visuo-spatial processing tasks, both hemispheres seem to contribute substantially [53]. Moreover, in a recent visual creativity study with a canonical right hemisphere task, the creativity task more strongly recruited left brain regions, while the control visuo-spatial task more strongly recruited right regions [63]. The current results indicate an important role of a widespread brain network involved in flexible visual processing of spatial relationships [64] in visuo-spatial divergent thinking.

### The role of age in the relation between gray matter morphology and divergent thinking

The observed relationship between gray matter morphology and visuo-spatial divergent thinking was independent of age, suggesting a general positive association between flexible visual processing of spatial relationships and cortical thickness and volume in a widespread brain network. Interestingly flexibility of verbal divergent thinking showed a negative relation between cortical thicknesses of the left SMG in adolescents only. A tentative but speculative explanation may be that a more mature SMG (as reflected by cortical thinning during mid adolescence; [16], [65]) is beneficial to flexible thinking. Yet, AUT-brick flexibility performance did not differ between adolescents and young adults. Moreover, brain activity during the AUT-scanner did not differ between adolescents and young adults [29]. Given the relative early maturation of the SMG as compared to prefrontal areas [16], [65], it would be interesting to see if the same association can be found in children and early adolescence.

### Strengths and future recommendations

The current study has a number of important strengths. First, the dual analyses approach allowed us to examine brain-behavior relationships with and without *a priori* assumptions about specific brain regions. Moreover, these “assumptions” were unbiased, as they were based on brain activations patterns derived from a functional study. Second, our population allowed us to investigate developmental effects of divergent thinking by comparing adolescents with young adults. Third, the use of a verbal and a visuo-spatial allowed us to examine different aspects of divergent thinking. Finally, we were able to integrate brain functional and structural measures to ensure a comprehensive understanding of the associations between divergent thinking and brain indices. A direct relationship between brain activity and structure is often assumed, but not always reported [66]. Even though BOLD activations during a verbal divergent thinking task did not significantly correlate with gray matter morphology, the combination of fMRI and MRI furthers our understanding of brain structure-function relationships and guides future hypotheses.

The psychometric properties of the CAT are good [11] and have been used previously in adolescents and adults [14]; however, the relation to other visuo-spatial divergent thinking tests (such as figural version of the Torrance Test for Creative Thinking, or mental rotation tasks) remains unknown. Future studies should demonstrate whether brain structure-function associations regarding the CAT generalize across the entire visuo-spatial domain of creativity.

An important difference between the current study and other structural MRI studies is the use of in-scanner behavioral measures (AUT-scanner) combined with divergent thinking measures outside the scanner (AUT-Brick and CAT). Only a categorical self-reported measure of AUT-scanner fluency was available for brain-behavioral analyses. Similarly as reported by Cousijn et al. [30], post-hoc analyses showed that answers on the AUT-scanner were correlated with AUT-brick fluency (*r* = .54) and with AUT-brick flexibility (*r* = .36), increasing the confidence of AUT-scanner fluency as a reliable measure of verbal divergent thinking. Moreover, the response distribution was relatively equal across the four AUT-scanner answer categories (i.e., 0–1, 2, 3, 4+), suggesting that these answer categories suffice in measuring AUT-fluency. Originality scores may be more informative for the creative process than fluency [67]. Indeed, Fink and colleagues [25] reported a positive association between AUT-originality in the right cuneus and occipital regions. However, originality and feasibility scores derived from our single-item AUT-Brick did not show an association with gray matter morphology. Furthermore, AUT-Brick performance was scored by a single trained rater. Even though extensive training resulted in high inter-rater reliabilities on the AUT-Brick in independent studies that were simultaneously conducted with the current study, a potential generalizability limitation of one rater cannot be excluded. In future studies, it is recommended to include a multi-item AUT outside the scanner. Moreover, although methodologically challenging, recording the alternative uses provided by the participants during scanning may provide more in depth knowledge about the neural mechanisms underlying divergent thinking.

### Conclusions

Divergent thinking is a complex cognitive construct. It has been operationalized in many different ways, which is reflected in the inconsistencies between neuroimaging studies investigating the neural mechanisms underlying divergent thinking (for reviews see; [2], [3], [4], [68]). Interestingly, our ROIs were based on a verbal divergent thinking paradigm, yet, we found an association between right MTG thickness and behavior on a visuo-spatial divergent thinking task. This suggests that the involvement of brain regions associated with the creative process is not limited to a specific domain. In future studies, it would be interesting to investigate the overlap between the neural network involved in visuo-spatial versus verbal divergent thinking in the same participants.

The literature investigating the relation between divergent thinking and gray matter morphology is relatively sparse, yet available findings are predominantly positive. To date, five studies, including our own, have showed positive associations between various divergent thinking measures and gray matter structural brain indices [22]–[25], and one study showed a mixture of both positive and negative associations depending on the creativity index used [26]. These findings tend to converge into the notion that greater gray matter volume or cortical thickness is associated with better computational efficacy of specific brain regions [31]. We should caution, however, against drawing any far-reaching conclusions, because not all aspects of divergent thinking show brain-behavior associations, and the associations vary in strength (see also [3]).

Our results offer a novel contribution to the understanding of the neuroanatomical basis of divergent thinking. Here we showed that a visuo-spatial measure (but not a verbal measure) of divergent thinking (CAT) was positively related to cortical thickness in occipital, parietal, temporal and frontal areas, independently of age. The results support an important role of a widespread brain network involved in flexible visual processing of spatial relationships in visuo-spatial divergent thinking. The current study provides the first evidence for a relation between cortical thickness and visuo-spatial divergent thinking in adolescents and young adults, taking an important step in unraveling the relationship between creative cognition and brain morphology.
